# Enhanced type I interferon signature in primary APS patients is associated with thrombocytopenia

**DOI:** 10.3389/fimmu.2026.1863789

**Published:** 2026-07-24

**Authors:** Sophie Scholz, Eduard Nitschke, Léa-Sophie Dreveton, Annika Wiedemann, Beniam Assefa, Franziska Szelinski, Eva Schrezenmeier, Axel Pruß, Gerhard Krönke, Thomas Dörner, Ana-Luisa Stefanski

**Affiliations:** 1 Department of Rheumatology and Clinical Immunology, Charité- Universitätsmedizin Berlin, Berlin, Germany; 2 Deutsches Rheumaforschungszentrum (DRFZ), Berlin, Germany; 3 Institute of Transfusion Medicine, Charité – Universitätsmedizin Berlin, Berlin, Germany; 4 Department of Nephrology and Intensive Medical Care, Charité- Universitätsmedizin Berlin, Berlin, Germany

**Keywords:** anti-phospholipid antibodies, anti-phospholipid syndrome, interferon, thrombocytopenia, thrombosis

## Abstract

**Introduction:**

Antiphospholipid syndrome (APS) is an autoimmune disorder characterized by thrombotic events and/or obstetric complications due to persistent antiphospholipid antibodies. Type I interferon (IFN) upregulation has been identified as a potential contributor to the pathophysiology of APS, however, the relationship between type I IFN and clinical manifestations of APS still needs to be delineated. The objective of this study was the evaluation of the potential role of type I IFN regarding different APS manifestations.

**Methods:**

We conducted a retrospective analysis of APS patients who presented to our clinic between 2017 and 2024, focusing on clinical manifestations, recurring events and serological profiles in relation to Siglec-1 expression on monocytes, as a surrogate marker of type I IFN signature.

**Results:**

Out of the 218 APS patients included in the study, 123 were diagnosed with primary APS (pAPS) and 95 with secondary APS (sAPS), the latter mostly SLE associated, showing a general higher type I IFN signature as the pAPS cohort. While no significant differences in Siglec-1 expression were observed across most APS manifestations (venous, arterial, mixed and obstetric events), significantly higher type I IFN activity was detected in APS patients with thrombocytopenia compared to those without (p = 0.0061). Moreover, we found an inverse correlation between platelet numbers and Siglec-1 values. Interestingly, the difference in type I IFN signature was confirmed in the pAPS cohort with thrombocytopenia (p = 0.0242). In sAPS, Siglec-1 expression did not differ significantly between patients with and without thrombocytopenia (p = 0.4664). With regard to serological and clinical characteristics, patients with thrombocytopenia exhibited a higher prevalence of triple positivity, elevated levels of anti-cardiolipin IgG antibodies and an increased incidence of recurrent and mixed (arterial/venous) thromboembolic events.

**Discussion:**

To conclude, the data indicate that type I IFN may be a relevant factor for the occurrence of thrombocytopenia in pAPS. Moreover, thrombocytopenia in APS was linked to increased prevalence of triple positivity, higher anti-cardiolipin IgG titres, and higher disease severity, underscoring the importance of closer monitoring in this subgroup. These results warrant validation in prospective studies and could support potential therapeutic targeting of type I IFN in a subset of APS patients.

## Introduction

Antiphospholipid syndrome (APS) is a systemic autoimmune disorder primarily associated with venous and/or arterial thromboembolic events, obstetric complications, and/or microvascular events in the presence of persistently elevated antiphospholipid antibodies (aPL, including lupus anticoagulant, anti-cardiolipin antibodies, and anti-β2-glycoprotein antibodies) (1). Triple aPL positivity is associated with the highest risk for clinical manifestations and disease burden (2). Even though the epidemiology of APS is poorly understood, the estimated incidence and prevalence are supposed to be ranging between 1 to 2 and 40 to 50 cases respectively per 100.000 (3). The disease is classified according to the newly adjusted 2023 ACR/EULAR criteria for APS, which encompass both clinical and laboratory criteria. Compared with the revised Saporo criteria from 2006 (4), new manifestations have been incorporated into the 2023 revised clinical APS criteria, including heart valve vegetations and/or thickening and thrombocytopenia (5). APS can be distinguished into a primary (pAPS) and secondary (sAPS) form, the latter occurring in association with other underlying systemic autoimmune diseases, most commonly systemic lupus erythematosus (SLE) (1). A rare, life-threatening complication is catastrophic antiphospholipid syndrome (CAPS), characterized by life-threatening, severe thrombotic complications, usually microvascular as well as large-vessel, that affects multiple organs simultaneously or over a short period of time (1, 6).

The complex pathophysiology of APS remains largely unexplored, including intricate interactions between genetic predisposition, infectious or other triggers that stimulate antiphospholipid antibody (aPL) production and culminate into thromboinflammation and initiation of disease (7). However, since the presence of aPL itself, even at medium-high titres, is not always associated with APS-related manifestations, the hypothesis of a “two-hit model” has been proposed (8). The activation of the complement system by anti-β2-glycoprotein antibodies, which also trigger neutrophil activation and the release of neutrophil extracellular traps (NETs) through a TLR4-dependent mechanism are elements of this hypothesis. This process subsequently leads to activation of B cells and differentiation of plasma cells/plasmablasts, enrichment of atypical memory B cell subsets (9) and can culminate into epitope spreading and autoantibody formation as part of the disease-related feed-forward loop (10).

An enhanced expression of type I interferon (IFN) responding genes has been linked to the pathogenesis of various systemic autoimmune disorders, like SLE (11), Sjögren’s disease (12) or myositis (13). Recent research has demonstrated an upregulation of type I IFN signature also in APS patients compared to healthy controls (14). Interestingly, IFN-I pathway activation is detectable even in some aPL-positive individuals lacking clinical APS manifestations, and progressively increases with clinical complexity, reaching highest levels in patients with sAPS and SLE (14). This signature has been validated for both pAPS and sAPS patients, suggesting a link between an elevated type I IFN signature and the pathomechanisms of APS, as another aspect of the “two-hit-model” (10). Gene expression of type I IFN is triggered through the plasmacytoid dendritic cell pathway, activated by aPL mediated stimulation of toll-like receptors (TLRs), including TLR7 and TLR9, and resulting in increased immune activation, inflammation, and autoantibody formation (10). The type I IFN score shows a positive correlation with anti-β2-glycoprotein antibodies of IgG or IgM subtype, while it is negatively associated with the use of hydroxychloroquine and age (14). However, the pathogenetic, clinical and therapeutic implications of type I IFN expression in APS still need to be delineated. Given the clinical heterogeneity of APS, effective stratification biomarkers are currently lacking. The objective of this study is to assess the potential role of type I IFN in relation to clinical and serological profiles in APS. Identifying type I IFN-driven signatures may enable patient stratification and support the development of more precise, targeted therapeutic approaches.

## Material and methods

### Study design and data collection

We performed a retrospective study including all patients with a confirmed diagnosis of APS, according to the 2023 classification criteria of EULAR/ACR (5), who presented at the Department of Rheumatology and Immunology at Charité University Hospital between 2017 and 2024. All APS patients were systematically assessed for evidence of an underlying systemic autoimmune disease, such as SLE, based on clinical findings and immunological markers, including ANA and anti-double-stranded DNA antibodies. SLE patients were classified according to 2019 EULAR/ACR SLE classification criteria (15). In cases with evidence for another defined systemic autoimmune disease, the diagnosis was refined to secondary APS. Otherwise, patients were classified as primary APS. Plasma samples were collected from all patients and stored after obtaining written informed consent (EA1/002/16, EA1/302/16, EA1/009/17, EA1/215/18, EA1/270/24). A total of 218 patients and 649 patient visits were collected.

Clinical data and individual blood values were extracted from medical records and electronic entries using the hospital electronic system accordingly. Clinical data included venous, arterial, and mixed thromboembolic events, as well as obstetric complications and thrombocytopenia. Mixed thromboembolic events were defined as the occurrence of both venous and arterial manifestations within the same patient’s clinical history. Due to the limited number of patients presenting exclusively with obstetric manifestations, no distinction was made regarding the additionally presence of venous, arterial thromboembolic events or other obstetric complications in this subgroup.

Thrombocytopenia was evaluated as an independent criterion in patients with primary APS and was defined according to the 2023 ACR/EULAR APS classification criteria as at least one documented occurrence of a platelet count under 130x10^9^/liter (5). Further, patients diagnosed with medication-induced/related or isolated immune thrombocytopenia were excluded from the cohort of individuals with thrombocytopenia.

Laboratory characteristics were evaluated by analyzing the APS autoantibody profile (lupus anticoagulant, anti-cardiolipin antibody IgG and IgM titres and anti-β2-glycoprotein antibody IgG and IgM titres – the laboratory maximum linear range cut-offs was 100 U/ml for anti-cardiolipin IgG as well as for anti-β2 glycoprotein IgG/IgM and 120 U/ml for anti-cardiolipin IgG), antinuclear antibody (ANA) titer, platelet number and Siglec-1 (CD169) expression on CD14+ monocytes, as an approved surrogate marker for type I IFN signature during routine patient’s follow-up (16).

### Analysis of human platelet antigen antibodies

To assess the presence of conventional anti-platelet autoantibodies in APS, we analyzed levels of IgG-antibodies targeting platelet factor 4 (PF4) using the Milenia^®^ QuickLine-HIT Latarel-Flow-Test and IgG-antibodies against human platelet antigens (HPA) located on glycoprotein IIb/IIIa, glycoprotein Ib/IX, glycoprotein Ia/IIa, glycoprotein IV and HLA class I antigens. This analysis was conducted using the PAK™-Lx bead-based assay integrated in the Luminex^®^ platform (17).

### Statistical analysis

Descriptive statistics were employed to characterize clinical and laboratory features of each patient, including venous, arterial, mixed and obstetric events, thrombocytopenia as well as the presence and titres of antiphospholipid antibodies. These characteristics provided the foundation for subsequent analyses when different patient subgroups were compared.

For the analysis of data between two groups, Mann-Whitney test was applied and p values <0, 05 were considered significant. For the analysis of data between more than two groups, Kruskal-Wallis test followed by Dunn’s test for multiple comparisons was applied and p<0, 05 was considered significant.

For correlation analyses, Spearman correlations were applied with a significance level set at p<0, 05. All statistical analysis and graphical illustrations were obtained by GraphPad Prism Version 9 software package (GraphPad software, San Diego, CA, USA).

## Results

### Clinical characteristics of the APS cohort and its subsets

A total of 218 patients diagnosed with APS were followed at the Department of Medicine/Rheumatology and Immunology at Charité University Hospital between 2017 and 2024 (detailed summary in **Table 1**). Among the overall cohort, 123 patients were classified as having primary APS (pAPS) and 95 with associated APS in the context of another defined autoimmune disease (sAPS), while the predominant underlying condition was SLE (95.79%). The median age of the overall cohort was 48 years, and 80.73% of the included patients were female. A total of 39.02% of patients with pAPS and 42.11% of those with sAPS exhibited triple antibody positivity. Regarding thromboembolic manifestations, the majority exhibited venous (38.21% of pAPS and 40% of sAPS patients) or arterial events only (25.2% of pAPS and 24.21% of sAPS patients), while mixed manifestations were experienced less often (13.82% in pAPS versus 16.84% of the sAPS cohort). 22.76% of the pAPS patients versus 18.95% in the sAPS cohort had obstetric manifestations. More patients with sAPS displayed thrombocytopenia compared with pAPS patients (33.68% versus 18.67%), which is in line with previous reports in SLE (18).

**Table 1:** Demographic and clinical characteristics of the APS cohort and the underlying primary and secondary subsets.

Total number of patients	Primary APS	Secondary APS
	123	95
Age		
Median (IQR)	48 (37-58)	48 (37-57)
Under 50	57 (46.34%)	54 (56.84%)
Between 50 and 69	52 (42.28%)	37 (38.95%)
>70	14 (11.38%)	4 (4.21%)
Sex		
Male (19.27%)	30 (24.39%)	12 (12.63%)
Female (80.73%)	93 (75.61%)	83 (87.37%)
Underlying coexisting disease	–	
Systemic lupus erythematodes	–	91 (95.79%)
Sjögren disease	–	1 (1.05%)
Systemic sclerosis	–	1 (1.05%)
Rheumatoid arthritis + other autoimmune disease (morbus crohn or myasthenia gravis)	–	2 (2.1%)
Medication		
Prednisolone	17 (13.82%)	48 (50.53%)
Median of dosis in mg (IQR)	5 (4-7, 5)	5 (4-5.75)
Hydroxychloroquine	13 (10.57%)	49 (51.58%)
Other immunosuppressants	12 (9.76%)	57 (60%)
Vitamine K antagonist	58 (47.15%)	41 (43.16%)
DOACs	31 (25.2%)	18 (18.95%)
Heparine	18 (14.63%)	13 (13.68%)
Platelet inhibitors (low dose aspirin or clopidogrel)	36 (29.27%)	27 (28.42%)
Triple positivity	48 (39.02%)	40 (42.11%)
Clinical manifestations		
Venous manifestations	47 (38.21%)	38 (40%)
Arterial manifestations	31 (25.2%)	23 (24.21%)
Mixed thromboembolic manifestations	17 (13.82%)	16 (16.84%)
Obstetric manifestations	28 (22.76%)	18 (18.95%)
Thrombocytopenia	23 (18.67%)	32 (33.68%)

In line with international guidelines, the majority of patients was treated with vitamin K antagonists followed by low dose aspirin. As expected, more sAPS compared with pAPS patients received immunomodulatory and/or suppressive therapies (detailed summary in **Table 1**).

### Higher type I IFN signature in sAPS

**Figure 1:**
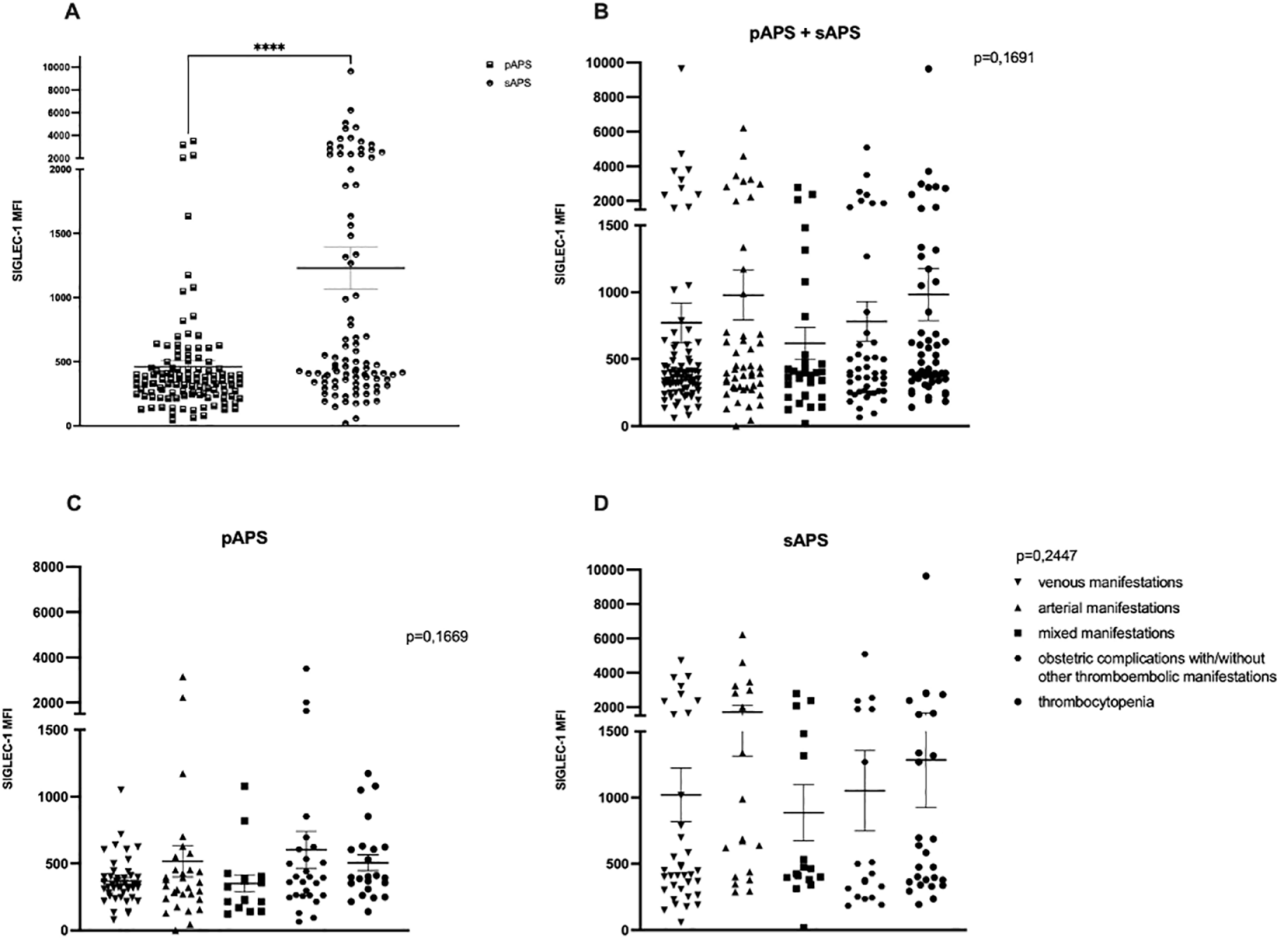
Siglec-1 (CD 169) expression on CD14+ monocytes of primary APS versus secondary APS patients **(A)**. Mean with SEM, Mann-Whitney test. Type I interferon signature detected by Siglec-1 expression on CD14+ monocytes in patients compared to different APS manifestations for **(B)** all APS diagnosis, **(C)** primary APS and **(D)** secondary APS. Mean with SEM, Kruskal-Wallis test followed by Dunn’s test.

A main aim of the study was to investigate potential associations between type I IFN and the various clinical APS manifestations as defined by the 2023 ACR/EULAR classification criteria. Siglec-1 is an established biomarker of the type I interferon (IFN) signature, validated in SLE, myositis, and infectious diseases (16), and is implemented as part of routine clinical diagnostics in our clinic. Since SLE, typically characterized by elevated type I IFN signature, was the main underlying systemic disease in our sAPS patients (95.8%), we compared initially the Siglec-1 expression levels between pAPS and sAPS cohorts. As expected, the analysis revealed significantly elevated Siglec-1 levels in sAPS compared to pAPS patients (p < 0.0001) (**Figure 1A**). An intriguing observation was the dichotomous distribution of Siglec-1 levels within the sAPS subgroup, mainly characterizing the heterogeneity of SLE and its disease activity, in line with previous reports (19).

### No association between different clinical manifestations and Siglec-1 expression

In a next step, we examined the relationship between Siglec-1 expression and distinct clinical manifestations of APS. Siglec-1 levels were compared among patients with venous thromboses, arterial thromboses, mixed thromboembolic events, obstetric complications and thrombocytopenia. This analysis was performed for the overall APS cohort, as well as separately for patients with pAPS and sAPS. There was a pronounced heterogeneity regarding Siglec-1 expression in all groups, however, we observed no significant differences between individual clinical manifestations (**Figures 1B–D**).

### Increased type I IFN signature in pAPS with thrombocytopenia

**Figure 2:**
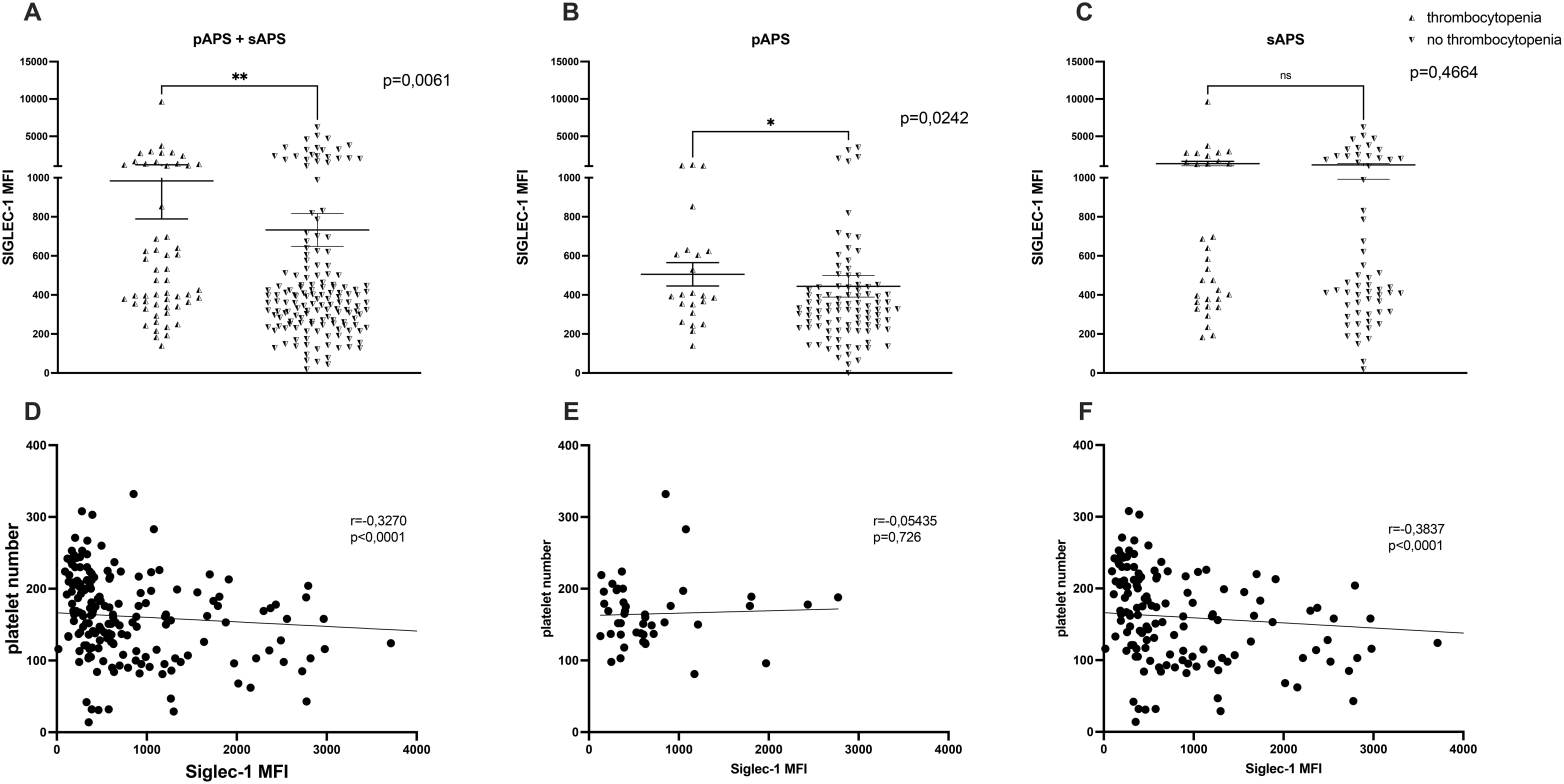
Type I interferon signature detected by Siglec-1 (CD 169) expression on CD14+ monocytes in patients with thrombocytopenia versus without thrombocytopenia for **(A)** all APS diagnosis, **(B)** primary APS and **(C)** secondary APS. Correlation between the total platelet count and Siglec-1 expression among **(D)** all APS patients, **(E)** primary APS patients and **(F)** secondary APS patients with thrombocytopenia. Mean with SEM, Mann-Whitney test, * for p<0, 05; ** for p<0, 01 [for **(A–C)**]. *Spearman correlation* [for **(D–F)**].

Subsequent analyses comparing the presence or absence of individual APS manifestations in relation to Siglec-1 expression showed no significant difference with respect to venous, arterial, mixed and obstetric manifestations (**Supplementary Figures 1A–L**). Most notably, however, the presence of thrombocytopenia (n=55) was associated with significant higher Siglec-1 expression levels compared to APS patients without thrombocytopenia (p = 0.0061; **Figure 2A**).

This finding was further confirmed in the subgroup of pAPS patients with thrombocytopenia (p = 0.0242, n= 23) (**Figure 2B**). In the sAPS cohort, the generally elevated Siglec-1 values did further differentiate patients with versus without thrombocytopenia (n= 32; **Figure 2C**).

A closer examination of the relationship between absolute platelet counts and Siglec-1 values revealed an inverse correlation between the two parameters (r = -0.3270, p < 0.0001) (**Figure 2D**). However, this relationship was not observed in the pAPS cohort (r = -0.05435, p = 0.726) (**Figure 2E**), likely due to the limited sample size, whereas a significant inverse correlation was confirmed for sAPS patients (r = -0.3837, p < 0.0001) (**Figure 2F**).

Interestingly, another subgroup analysis revealed also higher Siglec-1 values in the presence of arterial manifestations in the sAPS cohort only (p = 0.0485) (**Supplementary Figure 1J**).

### Higher prevalence of thrombocytopenia in male patients with pAPS

In a subsequent analysis, we evaluated demographic, clinical and serologic characteristics of APS patients with thrombocytopenia (**Table 2**).

**Table 2:** Demographic and clinical characteristics of the APS cohort with thrombocytopenia and without thrombocytopenia.

Total number of patients	APS with thrombocytopenia	APS without thrombocytopenia
	55	163
APS subgroup		
Primary APS	23 (41.82%)	100 (61.35%)
Secondary APS	32 (58.18%)	63 (38.65%)
Age		
Median (IQR)	48, 5 (35, 75-56, 25)	49 (37-58)
Under 50	30 (54.55%)	78 (47.85%)
Between 50 and 69	25 (45.45%)	66 (40.49%)
>70	2 (3.64%)	16 (9.82%)
Sex		
Male	11 (20%)	31 (19%)
Female	44 (80%)	132 (81%)
Underlying disease of secondary APS		
Systemic lupus erythematodes with or without one or more other autoimmune diseases	28 (87.5%)	63 (100%)
Sjögren syndrome	1 (3.13%)	0
Systemic sclerosis	1 (3.13%)	0
Rheumatoid arthritis + other autoimmune disease	2 (6.25%)	0
Medication		
Prednisolon	23 (41.82%)	42 (25.77%)
Hydroxychloroquin	17 (30.91%)	45 (27.61%)
Other immunosuppressants	25 (45.45%)	43 (26.38%)
Vitamin k antagonist	27 (49.09%)	73 (44.79%)
DOAC	16 (29.09%)	35 (21.47%)
Heparin	6 (10.91%)	24 (14.72%)
Platelet aggregation inhibitors (low dose aspirin or clopidogrel)	14 (25.45%)	48 (29.45%)
Triple positivity	30 (54.55%)	58 (35.57%)
Clinical manifestations		
Venous manifestations	21 (38.2%)	64 (39.5%)
Arterial manifestations	12 (21.8%)	41 (25.3%)
Mixed thromboembolic manifestations	12 (21.8%)	21 (13%)

**Figure 3:**
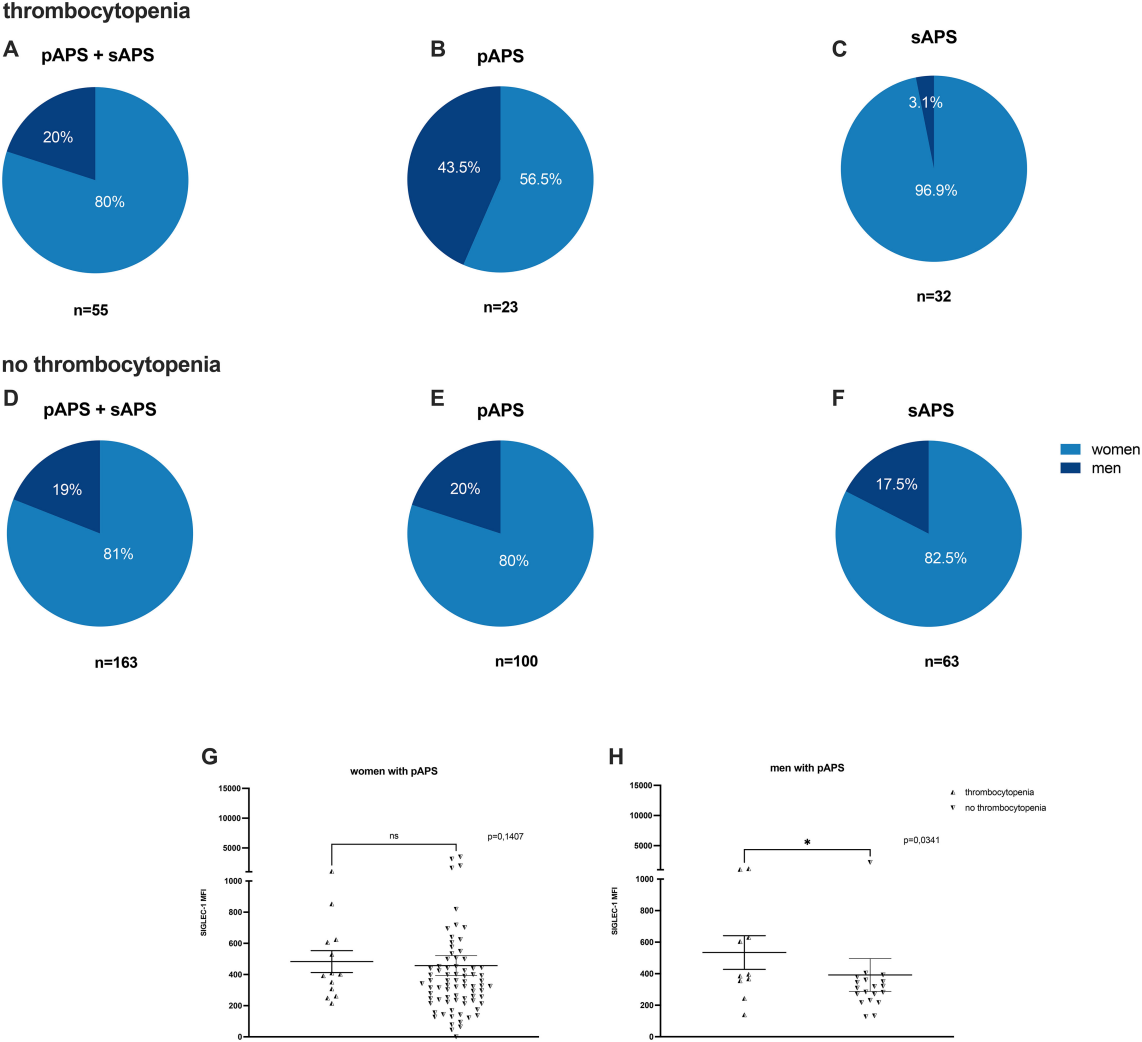
Gender distribution in APS patients in the presence **(A–C)** or absence **(D–F)** of thrombocytopenia. **(A)** and **(D)** show all APS patients, **(B)** and **(E)** show primary APS patients and **(C)** and **(F)** show secondary APS patients. Type I interferon signature detected by Siglec-1 expression on CD14+ monocytes in pAPS patients with thrombocytopenia versus without thrombocytopenia **(G)** for women and **(H)** for men. Mean with SEM, Mann-Whitney test, *for p<0, 05; **for p<0, 01..

Despite the significantly higher prevalence of female patients in the overall APS group (**Supplementary Figures 2A–C**), thrombocytopenia occurred in the pAPS cohort with a more than 10fold increased prevalence among male patients than within the sAPS cohort. (43.5% versus 3.1%; **Figures 3A–F**). Interestingly, male patients with thrombocytopenia in pAPS presented higher Siglec-1 values when compared with non-thrombocytopenic pAPS patients (**Figures 3G, H**).

### APS patients with thrombocytopenia experience higher frequency of mixed and recurrent thromboembolic events

**Figure 4:**
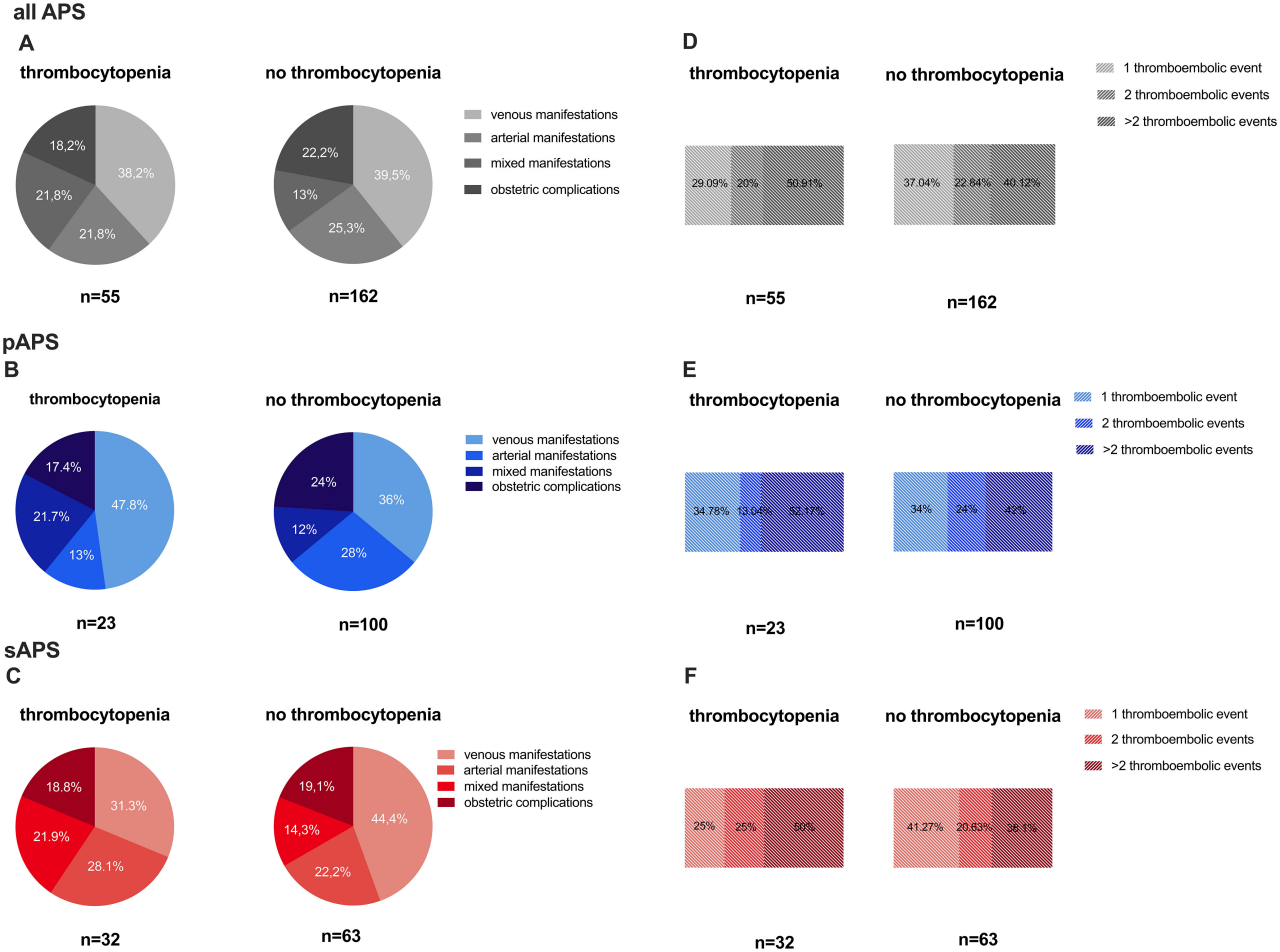
Distribution of clinical manifestations regarding presence or absence of thrombocytopenia in all APS patients **(A)**, in patients with primary APS **(B)** and secondary APS **(C)**. Recurrence of thromboembolic manifestations with versus without thrombocytopenia for all APS patients **(D)**, patients with primary APS **(E)** and secondary APS **(F)**.

To assess further clinical manifestations in APS patients with thrombocytopenia, we compared the distribution of various vascular manifestations of APS, including venous, arterial, mixed, as well as obstetric manifestations. As a result, we observed a higher frequency of mixed venous and arterial manifestations in APS patients with thrombocytopenia, as compared to those without (21.8% vs. 13%) (**Figure 4A**). This finding was confirmed by subsequent analyses for both subgroups, pAPS and sAPS (**Figures 4B, C**). Regarding the other manifestations, pAPS patients with thrombocytopenia experienced more venous (47.8% versus 36%) and less arterial (13% versus 28%) and obstetric events (17.4% versus 24%), while secondary APS patients with thrombocytopenia had a higher prevalence of arterial manifestations (28.1% versus 22.2%; **Figure 4C**) compared with the cohorts without thrombocytopenia.

Since recurrent events represent a more severe clinical phenotype and a higher disease burden, we evaluated this aspect in our cohort. Our analysis revealed that the majority of the patients experienced two or more thromboembolic events, with even a higher prevalence in the thrombocytopenia group (70.91% with thrombocytopenia versus 62.96% without thrombocytopenia (**Figure 4D**). This finding was corroborated by subsequent analysis in both subgroups, pAPS and sAPS (**Figures 4E, F**). Noteworthy, the highest prevalence of recurrent events—and thus the highest disease burden—was observed in sAPS patients with thrombocytopenia (72.6%).

### Thrombocytopenia is associated with triple-positivity and higher IgG anti-cardiolipin-antibody levels

**Figure 5:**
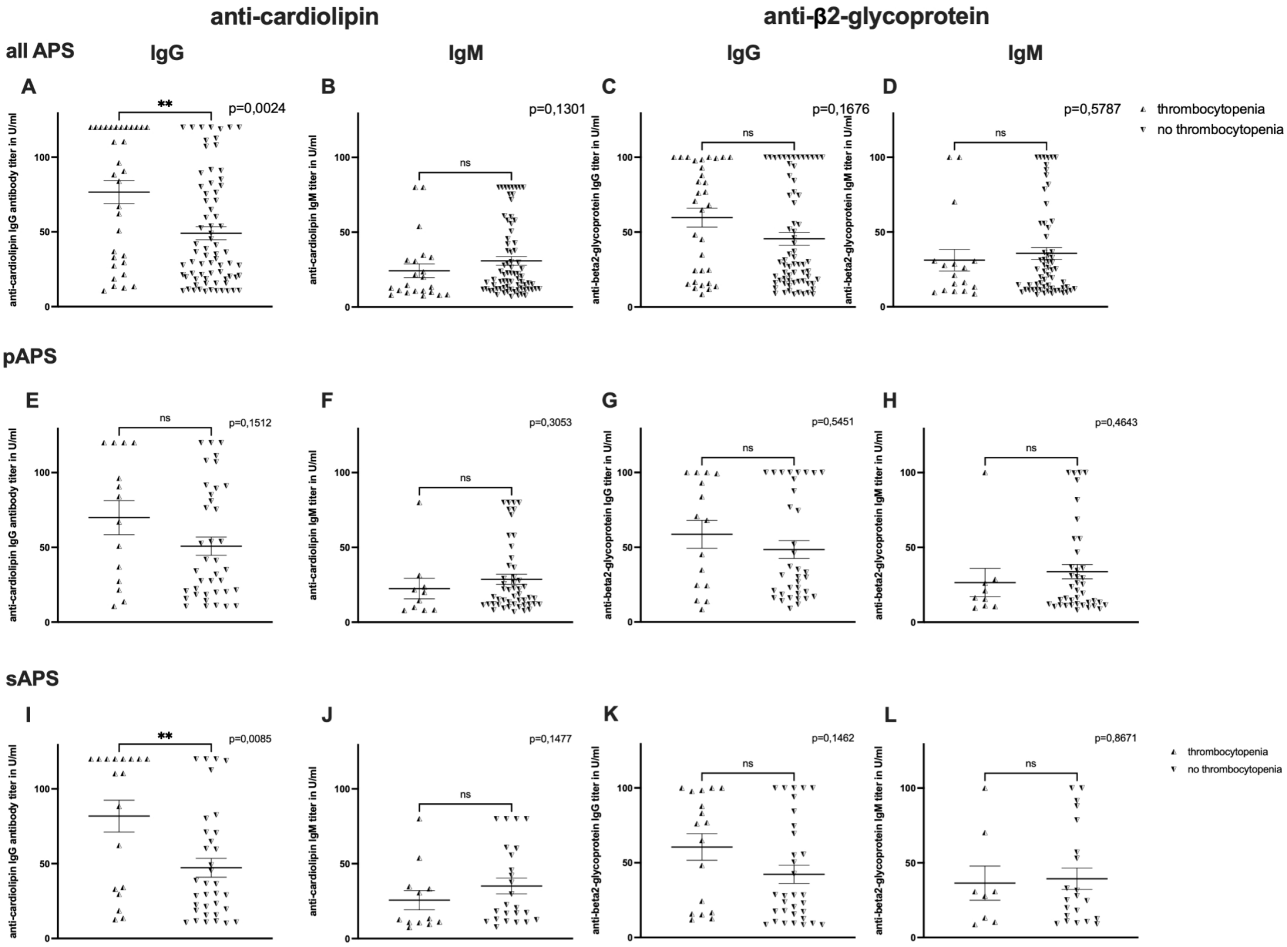
Antibody titres and the presence/absence of thrombocytopenia compared between all APS patients **(A–D)**, primary APS patients **(E–H)** and secondary APS patients **(I–L)** with versus without thrombocytopenia for anti-cardiolipin IgG **(A, E, I)**, anti-cardiolipin IgM **(B, F, J)**, anti-β2-glycoprotein IgG **(C, G, K)** and anti- β2-glycoprotein IgM **(D, H, L)**. *Mean with SEM, Mann-Whitney test*.

In a next step we assessed serologic characteristics of the APS cohort with thrombocytopenia. In general, the presence of thrombocytopenia was associated with higher prevalence of triple positivity [54.55% versus 35.57% (**Table 2**)]. Furthermore, APS patients with thrombocytopenia exhibited significantly higher titres of IgG anticardiolipin antibodies compared to those without thrombocytopenia (p=0.0024) (**Figures 5A–D**), which was not confirmed in the pAPS (**Figures 5E–H**) but in the sAPS subgroup (p = 0.0085; **Figures 5I–L**). No significant differences were found for the IgM subtype of anticardiolipin antibodies, nor for the IgG and IgM subtypes of anti-β2-glycoprotein antibodies (**Figures 5A–L**). Looking into the total APS cohort, no associations could be found between Siglec-1 expression levels and autoantibody titres (**Supplementary Figure 3**).

### No significant relationship between human platelet antigen antibodies and thrombocytopenia in APS

Finally, we investigated whether certain anti-human platelet antigen antibodies may be potentially involved in the observed thrombocytopenia, such as anti-platelet factor 4 (PF4) antibodies, anti-HLA class I antibodies and different antibodies against platelet glycoproteins (GP IIb/IIIa, GP Ib/IX, GP Ia/IIa, and GP IV). Here, only few patients showed detectable autoantibodies independently of thrombocytopenia and thus did not provide a consistent profile (**Supplementary Table 1**). Together, these serologic findings do not support the hypothesis of a bystander or cross-reactive activation of certain anti-platelet autoantibodies in APS typically involved in the occurrence of thrombocytopenia.

## Discussion

This study investigated the relationship between laboratory and clinical characteristics of APS patients with respect to increased type I IFN. We could show for the first time that an elevated type I IFN signature is associated with the presence of thrombocytopenia in APS and correlates inversely with total platelet counts. Moreover, the APS patient subgroup with thrombocytopenia presented a notably higher prevalence of triple positivity and substantially higher anticardiolipin IgG antibody titres together with a higher disease burden by increased occurrence of mixed and recurrent thromboembolic events. These findings indicate a potential association with disease severity in this subset of APS patients, requiring closer monitoring and supporting further investigation of targeted therapeutic interventions.

Thrombocytopenia, a newly included criterion in the 2023 ACR/EULAR APS classification (5) is respectively defined as a platelet count of 20–130 Gpt/L and experienced at least transient by round one fourth of all APS patients (20). Platelets play a crucial role in APS pathophysiology as cellular player at the intersection of inflammation and coagulation, possessing a vast array of immune receptors and ligands that drive immune activation and sustain thrombus formation (21). Their immunological activity is involved in various mechanisms, by activating the complement system (22), TLRs (23), or dendritic cells via CD40L (24), the later further promoting IFN I production and sustaining the inflammatory response (25).

First mechanistic links between type I IFN and thrombocytopenia were described following different viral infections (26). Additionally, mild thrombocytopenia is a common adverse effect in exogeneous administration of pegylated interferon-alpha, as former standard therapy for chronic hepatitis C (27). This effect is primarily mediated by late-stage inhibition of megakaryocytes maturation and reduction of platelet output form the bone marrow via downregulation of transcription factors like GATA-1 (28). IFN-alpha therapy can rarely also trigger severe immune-mediated thrombocytopenia, characterized by increased platelet-associated IgG and responsiveness to immunosuppressive therapies (29). Finally, elevated type I IFN signature is also observed in immune thrombocytopenic purpura (ITP), indicating a role in immune-mediated platelet activation and consumption (30).

In the context of APS, thrombocytopenia is considered a multifactorial and incompletely understood manifestation. Proposed mechanisms include aPL-mediated platelet activation and peripheral destruction via anti-β2GPI/β2GPI complexes (31), immune-mediated platelet clearance (32), cytokine-driven alterations in bone marrow megakaryopoiesis as well as complement and NET activation resulting in accelerated clearance (31). In line with this, IFN-I may further amplify thromboinflammation by inducing aPL production (33–35),, while aPL can activate plasmacytoid dendritic cells and promote additional IFN-I release as feed-forward loop (25). Notably, our findings do not support the hypothesis of a bystander or cross-reactive activation of conventional anti-platelet autoantibodies such as anti-PF4 antibodies in APS patients with thrombocytopenia. However, the potential contribution of assay-related limitations cannot be entirely excluded.

Clinically, similar to our data, Shi et al. described the presence of thrombocytopenia in association with a higher disease burden and occurrence of other severe extra-criteria manifestations (21). Moreover, the presence of aPL seems to increase the risk of thrombocytopenia in SLE patients (36). Our analysis suggested that male pAPS patients with enhanced IFN-I signatures may exhibit higher rates of thrombocytopenia and greater disease burden. However, the underlying mechanisms remain uncertain and may involve sex-specific immune regulation, hormonal influences, or stochastic subgroup effects, necessitating independent validation in larger cohorts. In addition, secondary APS patients with thrombocytopenia, predominantly associated with SLE, showed the highest thrombotic burden, strongest aPL responses, and most pronounced IFN-I activation. Given the substantial contribution of SLE to these clinical outcomes, it is difficult to determine whether the observed associations are driven by APS-specific mechanisms or by overlapping pathophysiological pathways common to both APS and SLE.

Our findings support the view that APS patients with thrombocytopenia represent a more severely affected subgroup characterized by increased immunological and thrombotic burden. Whether this subgroup represents a distinct biological endotype or reflects greater overall disease severity remains to be determined. However, given the potential link between type I IFN, thrombocytopenia, and disease severity, biomarker-driven patient stratification may enable therapeutic strategies beyond anticoagulation. Potential immunomodulatory options with effects upon type I type IFN activity include hydroxychloroquine (36) as already been used in patients with recurrent events. Prospective studies are required to determine the utility of targeted interventions in APS-associated thrombocytopenia like hydroxychloroquine and monoclonal antibodies targeting IFN or its receptors (36).

Our study reports on a large APS cohort with long-term clinical and laboratory data; however, some limitations require consideration. The retrospective character and limited patient numbers in some subgroups (esp. with obstetric complications) reduced the statistical power of some analyses. Furthermore, the assessment of antibody titres was constrained by laboratory maximum linear range cut-offs and longitudinal IFN trajectories are not available. In addition, in sAPS patients potential confounding due to overlapping pathophysiological pathways common to both APS and SLE or immunomodulatory treatment must be considered. Although Siglec-1 is a well-established and validated surrogate marker of type I IFN activity across multiple systemic autoimmune diseases (16), it does not directly quantify circulating type I IFN levels.

This study demonstrates for the first time that an elevated type I IFN signature is associated with thrombocytopenia in APS. APS patients with thrombocytopenia represent a distinct subgroup characterized by higher disease burden, including increased prevalence of triple positivity, elevated anticardiolipin IgG titres, and greater risk of mixed and recurrent thromboembolic events. Secondary APS patients with thrombocytopenia showed the most pronounced disease severity typically related to SLE, highlighting the need for closer monitoring and tailored therapeutic strategies. Our findings suggest that type I IFN could contribute to the pathophysiology of thrombocytopenia in APS, potentially through multifactorial mechanisms. This underscores the rationale for exploring immunomodulatory therapies in addition to standard anticoagulation, including agents like hydroxychloroquine known to downregulate IFN production. Prospective studies are needed to validate these findings and to assess the efficacy of targeted interventions for APS patients with thrombocytopenia.

## Data Availability

The original contributions presented in the study are included in the article/**Supplementary Material**. Further inquiries can be directed to the corresponding author.
