# Supplementary material for: Enhanced type I interferon signature in primary APS patients is associated with thrombocytopenia
Source: Front Immunol. 2026 Jul 24;17:1863789. doi: 10.3389/fimmu.2026.1863789 (PMC13447253; doi:10.3389/fimmu.2026.1863789)
Supplement: Supplementary file 1 [file DataSheet1.docx]

**Fig.1** Type I interferon signature (Siglec-1 expression on Cd14+ monocytes) in **(A-D)** all APS **(E-H)** pAPS and **(I-L)** sAPS patients comparing the occurrence and absence of **(A, E, I)** venous manifestations, **(B, F, J)** arterial manifestations, **(C, G, K)** mixed (arterial and venous) manifestations and **(D, H, L)** obstetrical manifestations APS. *Mean with SEM, Mann-Whitney test, * for p<0,05; ** for p<0,01.*

**Fig.2** Gender distribution in APS patients for **(A)** all APS patients, **(B)** pAPS patients and **(C)** sAPS patients.

Type I Interferon signature as captured by Siglec-1 expression on monocytes in patients with thrombocytopenia versus without thrombocytopenia **(D,E)** for women and **(F,G)** for men showing **(D,F)** all APS diagnosis and **(E,G)** secondary APS. *Mean with SEM, Mann-Whitney test, * for p<0,05; ** for p<0,01.*

**Fig. 3:** Spearman´s correlation matrix showing relation between Siglec-1 values, platelet number and aPL titer for all APS patients. Corresponding correlations are represented by red (negative) or blue (positive) colour. Values in circles indicates r value of correlation.

**Table 1**: Occurrence and frequency of human platelet antigen antibodies in APS patients with versus without thrombocytopenia

|  | **Thrombocytopenia (n=55)** | **no thrombocytopenia (n=163)** |
| --- | --- | --- |
| Anti-platelet factor 4 (PF4) antibodies | 1 (1.82%) | 3 (1.84%) |
| Anti-HLA class I antibodies | 3 (5.45%) | 3 (1.84%) |
| Anti-GP IIb/IIIa antibodies | 2 (3.64%) | 0 |
| Anti-GP Ib/IX antibodies | 0 | 0 |
| Anti-GP Ia/IIa antibodies   - Anti-HPA-5b antibodies | 1 (1.82%) | 0 |
| Anti-GP IV antibodies | 0 | 0 |
